# Enhancement of memory and emotional functions by long-term ingestion of protease-treated porcine liver extract in mice

**DOI:** 10.1038/s41598-025-03362-4

**Published:** 2025-05-28

**Authors:** Taiga Kurihara, Masanori Sasanuma, Masahiro Kato, Toshio Inoue, Naoko Okada, Teruki Shirayama, Yusuke Tanikawa, Taiki Kawahara, Kazuhiro Sohya, Hiroki Yasuda, Yoshikazu Matsuda, Takeshi Uemura

**Affiliations:** 1 Division of Physiology, Faculty of Medicine, Saga University, Saga, 849-8501 Japan; 2 Division of Microbiology and Molecular Cell Biology, Nihon Pharmaceutical University, Saitama, 362-0806 Japan; 3 Division of Pharmacotherapy, Nihon Pharmaceutical University, Saitama, 362-0806 Japan; 4 Department of Biomedical Engineering, Graduate School of Medicine, Science and Technology, Shinshu University, Nagano, 390-8621 Japan; 5 Department of Orthopedic Surgery, Shinshu University School of Medicine, Nagano, 390-8621 Japan; 6 Division of Clinical Pharmacology and Pharmaceutics, Nihon Pharmaceutical University, Saitama, 362-0806 Japan; 7 Division of Gene Research, Research Center for Advanced Science and Technology, Shinshu University, Nagano, 390-8621 Japan; 8 Institute for Biomedical Sciences, Research Cluster for Social Implementation, Shinshu University, Nagano, 390-8621 Japan

**Keywords:** Porcine liver, Nutrition, Memory, Anxiety, Locomotor activity, Animal behaviour, Nutritional supplements

## Abstract

The relationship between diet and brain functions has garnered attention. Previous studies have shown that ingesting a protease-treated porcine liver decomposition product (PLDP) improves cognitive function in humans. In this study, we investigated the effects of PLDP ingestion on cognitive and emotional functions in mice. Mice were fed a PLDP-enhanced diet for 16 weeks and subjected to various behavioral assessments. PLDP ingestion enhanced long-term memory in Barnes maze test. Moreover, mice fed the PLDP diet exhibited reduced anxiety- and depression-like behaviors as evidenced by their performance in open-field, elevated plus maze, marble-burying, and forced swim tests. They also increased locomotor activity. RNA sequencing analysis of the brain tissue revealed substantial changes in gene expression, particularly in pathways associated with learning, memory, and anxiety regulation. Collectively, these results suggest that PLDP induces changes in gene expression associated with brain function, potentially contributing to the enhancement of cognitive function and psychological health. Furthermore, our findings not only enhance our understanding of the relationship between nutrition and brain function but also indicate the potential of interventions utilizing dietary components, such as PLDP, to support cognitive function and psychological health.

## Introduction

Nutrition not only fulfills the body’s basic energy requirements but also plays a vital role in regulating brain health. In recent years, the intricate relationship between diet and brain function, including cognitive ability and emotional health, has garnered attention^1–4^. Numerous studies have highlighted the pivotal roles of various nutrients, including vitamins, choline, omega-3 fatty acids, flavonoids, minerals, and amino acids, such as tryptophan and tyrosine, in supporting cognitive health and mitigating mental health issues such as anxiety and depression^2,4,5^. The identification of dietary and nutritional components that influence brain function and behavior can provide insights into potential dietary interventions for cognitive and emotional disorders, as well as suggest novel pharmacological targets.

The liver is highly nutritious and has long been recognized as a valuable ingredient for nutritional supplementation. As a highly nutrient-dense organ, the dietary liver has exceptional nutritional value. It is abundant in high-quality proteins and contains essential nutrients, including vitamins such as folic acid, vitamin A, and vitamin B^6,7^. Furthermore, the liver provides important nutrients such as fat-soluble antioxidant coenzymes Q10, choline, and taurine^8–10^.

Protease-treated porcine liver decomposition product (PLDP) is obtained through the enzymatic hydrolysis of porcine liver proteins^11^. Our previous human clinical trials involving 10 to 50 participants suggest that long-term ingestion of PLDP improves cognitive function, particularly memory and recall abilities^11–13^. Specifically, participants ingested PLDP orally for four weeks have shown improved scores in visual memory and delayed recall assessments using the Revised Hasegawa Dementia Scale and Wechsler Memory Scale-Revised, suggesting that ingestion of PLDP may positively affect cognitive function^11,12^.

In this study, we investigated the long-term effects of PLDP ingestion on cognitive and emotional functions in mice. Mice were fed a diet containing PLDP for 16 weeks and subjected to a series of behavioral tests for assessing memory, locomotor activity, anxiety, and depression. Additionally, RNA sequencing (RNA-seq) analysis was conducted on brain tissues to identify changes in gene expression related to PLDP ingestion.

## Results

### Ingestion of PLDP enhances long-term memory in mice

**Fig. 1:**
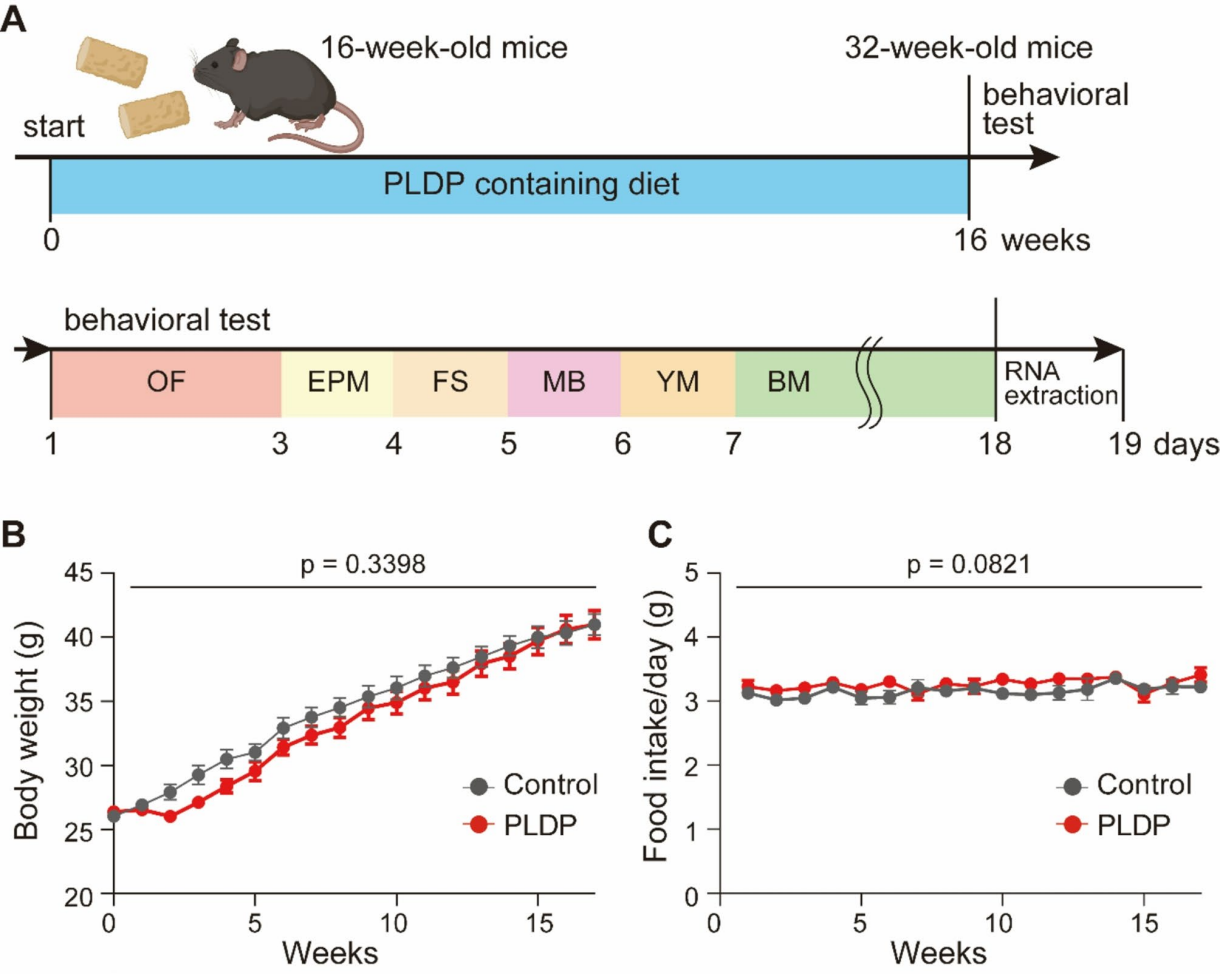
Schematic of the experimental design for evaluating the effects of PLDP ingestion in mice. (**A**) Sixteen-week-old male C57BL/6N mice were fed a PLDP-containing diet (PLDP mice) or a control diet (control mice) for 16 weeks, and behavioral analyses were conducted. One day after completing behavioral analyses, total RNA was extracted from their brains. The schema presents the timeline of PLDP diet feeding, behavioral analyses, and subsequent RNA extraction. EPM, elevated plus maze; FS, forced swimming; MB, marble burying, OF, Open field; YM, Y-maze. The graphic was created with Biorender.com. (**B**) Weekly changes in the body weights of the PLDP and control mice. Body weight was measured weekly. Two-way repeated measures ANOVA revealed no significant effect of diet on body weight (F(1, 24) = 0.9485, *p* = 0.3398, n = 13 each). (**C**) Food intake of the PLDP and control mice. The daily food intake of the PLDP and control mice was measured weekly. Two-way repeated measures ANOVA revealed no significant effect of diet on food intake (F(1, 24) = 3.293, *p* = 0.0821, n = 13 each). Data are represented as mean ± SEM.

**Fig. 2:**
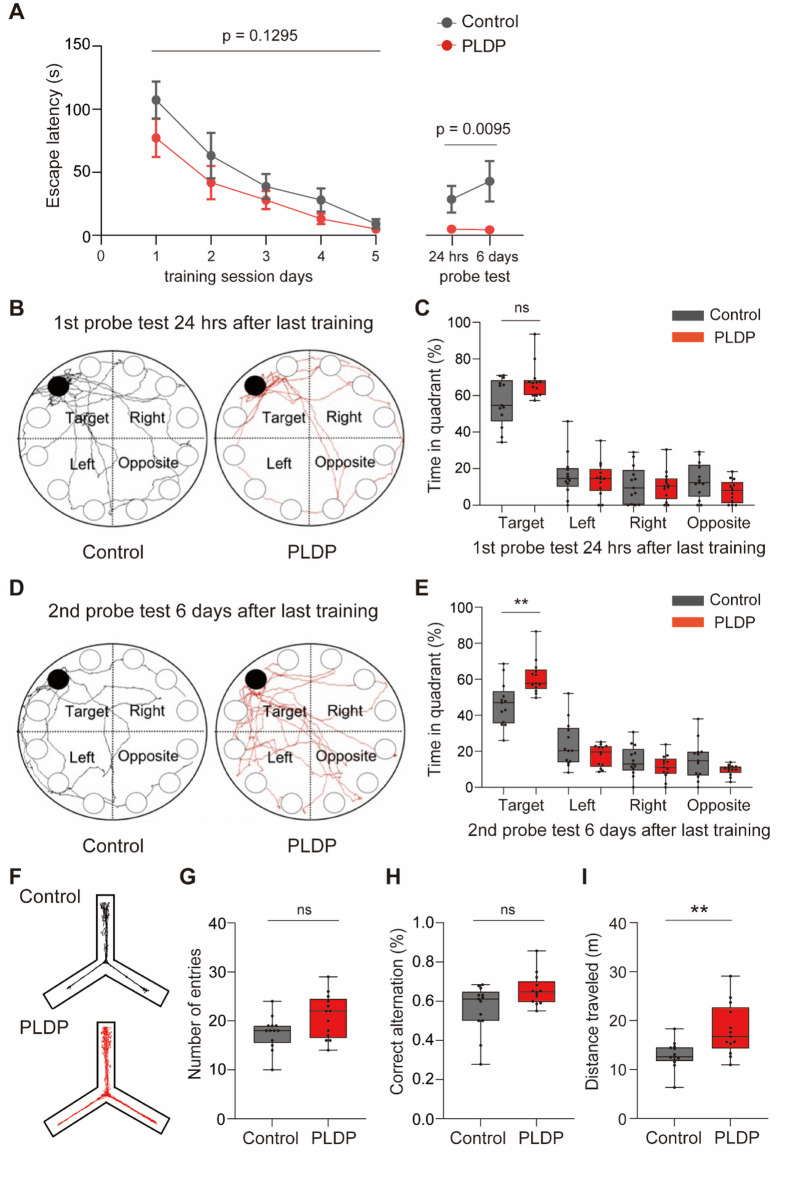
Ingestion of PLDP enhances memory and locomotor activity. (**A**–**E**) Barnes maze test. (**A**) Time taken to reach the target hole during the training session and probe tests. There was no significant difference in escape latency during the session between control and PLDP mice (two-way repeated measures ANOVA, n = 13 each, F(1,24) = 2.465, *p* = 0.1295). PLDP mice showed significantly shorter escape latency than control mice during the probe session (two-way repeated measures ANOVA, n = 13 each, F(1,24) = 7.950, *p* = 0.0095). (**B** and **D**) Representative trajectories of the PLDP and control mice in the 1st **(B)** and 2nd **(D)** probe tests. **(C** and **E**) Percentage of time spent in each quadrant during the 1st (**C**) and 2nd (**E**) probe tests. (**F–I**) Y-maze test. (**F**) Representative trajectories of the PLDP and control mice. **(G)** Total number of entries into the arms. (**H**) Percentage of correct alternations. **(I)** Distance traveled during the test. Data are represented as mean ± SEM in **(A)** and boxplots in **(C**, **E**, and **G**–**I)**. The horizontal line in each box shows the median; the box shows the interquartile range (IQR); and the whiskers are 1.5 × IQR. n = 13 in each group. **p < 0.01, *p < 0.05; Mann–Whitney’s U–test in **(C**, **E**, and **G**–**I**).

To examine the consequences of long-term ingestion of PLDP on learning and memory in C57BL/6N male mice, we fed them a specialized diet containing PLDP for 16 weeks and conducted a behavioral test (Fig. 1A). The composition of PLDP is shown in Table 1. The PLDP-containing diet was formulated according to the AIN-93 M guidelines^14^, while AIN-93 M diet with an adjusted total caloric content (Table 2) was used as the control. No significant differences in food intake or body weight were observed between mice fed the PLDP-containing diet (PLDP mice) and those fed the control diet (control mice) (Fig. 1B and C). We performed the Barnes maze test to assess spatial learning and memory capabilities^15^. During the training session from days 1 to 5, the PLDP and control mice demonstrated a learning curve, as evidenced by the decreasing escape latency (Fig. 2A). No significant differences were observed between the two groups during the acquisition phase (*p* = 0.1295). In probe tests conducted 24 h (1st probe test) and 6 days (2nd probe test) after the last training session, the PLDP mice exhibited significantly shorter escape latencies than the control mice (*p* = 0.0095) (Fig. 2A). In the 1st probe test, both groups showed a high preference for the escape target quadrant, with no significant differences between the groups (Fig. 2B and C). However, in the 2nd probe test, the PLDP mice exhibited a significantly higher percentage of time spent in the escape target quadrant than the control mice (*p* = 0.0019) (Fig. 2D and E). These results suggested that PLDP enhances long-term memory.

**Table 1:** Nutritional Composition of PLDP (per 100 g).

Component	Content	Component	Content
Carbohydrates^a^	53.3 g	Ash^g^	1.7 g
Proteins^b^	32.3 g	Moisture^h^	5.9 g
Peptides^c^	7.89 g	Minerals^i^	
Free amino acids	1.34 g	K	508 mg
Fats^d^	6.8 g	P	69.2 mg
Phospholipids^e^		Ca	46.1 mg
Phosphatidylcholine	1.91 g	Na	39.0 mg
Lysophosphatidylcholine	0.298 g	Fe	19.1 mg
Phosphatidylethanolamine	0.226 g	Zn	12.3 mg
Phosphatidylinositol	0.0947 g	Cu	2.4 mg
Sphingomyelin	0.0673 g	Mn	0.2 mg
Phosphatidic acid	0.057 g	Vitamins^j^	
Phosphatidylserine	0.00748 g	Vitamin A^h^	28.0 μg
Nucleic acids^f^		Vitamin B1^h^	0.4 mg
5'-Adenylic acid	51 mg	Vitamin B2^h^	4.9 mg
5'-Inosinic acid	39 mg	Vitamin B6^j^	0.7 mg
5'-Guanylic acid	26 mg	Vitamin K^h^	2.0 μg

^a^The value obtained by subtracting protein, fat, moisture, and ash from 100 g, which includes dextrin added as an excipient.

^b^The protein content was determined by combustion method.

^c^The peptide content was calculated as the measured amount of free amino acids subtracted from the value measured by HPLC after removing and neutralizing the protein with 5% perchloric acid.

^d^Measured by acid hydrolysis method.

^e^Phospholipids content was measured by Suzuki et al. 2024.

^f^HPLC method.

^g^Direct ashing method.

^h^Drying method.

^i^ICP emission Spectroscopy or atomic absorption spectrophotometry.

^j^Microbial Assay.

**Table 2:** Amounts of additives to the standard AIN-93 M diet and PLDP containing diet.

Ingredients	modified AIN-93 M diet	PLDP containing diet
PLDP (g)	–	20.0
casein (g)	14.0	3.5
cystine (g)	0.045	0.18
β-cornstarch (g)	46.6	46.6
α-cornstarch (g)	6.635	15.5
sucrose (g)	10.0	10.0
soybean oil (g)	4.0	3.5
cellulose powder (g)	5.0	5.0
AIN-93M mineral mix^a^ (g)	3.5	3.5
AIN-93 vitamin mix^b^ (g)	1.0	1.0
choline bitartrate (g)	0.25	0.25
tert-butylhydroquinone (g)	0.0008	0.0008
total (g)	100.0	100.0
total calories (kcal)	353.5	355.8

^a^Mineral mixtures designed for the maintenance of adult animals.

^b^Mixtures to supplement the vitamins required for adult animals.

Next, we evaluated the spatial working memory of mice by examining their spontaneous alternation behavior in a Y-maze^16^. This test assesses the spatial working memory based on the innate curiosity of mice to explore previously unvisited areas. No significant differences were observed in the total number of arm entries and the percentage of correct alternations between the two groups (Fig. 2F–H). Conversely, the PLDP mice exhibited a significant increase in the total distance traveled during the test period (*p* = 0.0029) (Fig. 2I). This result suggested that PLDP ingestion increases locomotor activity.

### Ingestion of PLDP enhances locomotor activity and reduces anxiety-like behaviors in mice

**Fig. 3:**
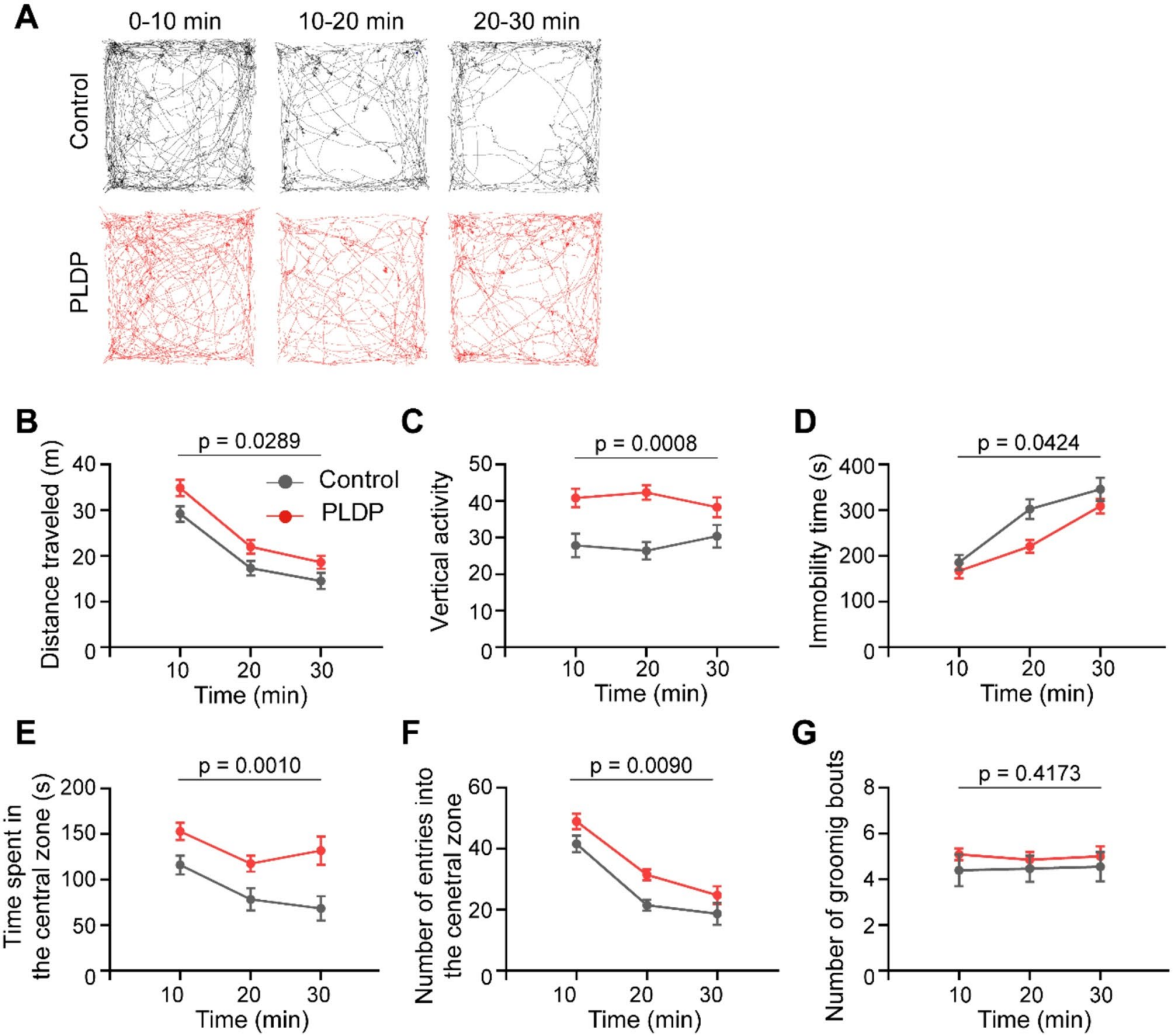
Ingestion of PLDP enhances locomotor activity and reduces anxiety-like behaviors. (**A**) Representative trajectories of the PLDP and control mice in the open field test. The trajectories at 10-min intervals are shown. (**B**) Distance traveled, (**C**) vertical activity, (**D**) immobility time, (**E**) time spent in the central zone, **(F)** number of entries into the central zone, and **(G)** number of grooming bouts at 10-min intervals. Data are represented as mean ± SEM. n = 13 in each group. The effects of the PLDP diet were analyzed using two-way repeated measures ANOVA; F(1,24) = 5.401; *p* = 0.0289 in (**B**), F(1,24) = 14.85; *p* = 0.0008 in (**C**); F(1,24) = 4.596, *p* = 0.0424 in (**D**); F(1,24) = 13.97, *p* = 0.001 in (**E**); F(1,24) = 8.072, *p* = 0.009 in (**F**) ; and F(1,24) = 0.6812, *p* = 0.4173 in (**G**). Statistical significance was analyzed by two-way repeated measures ANOVA.

We examined locomotor activity in an open-field test^17^. The PLDP mice exhibited significant increases in total travel distance, and vertical activity (quantified by number of rearing), along with a decrease in immobilization, compared to those of the control group (*p* = 0.0289, 0.0008, and 0.0424, respectively) (Fig. 3A–D), indicating increased locomotor activity. Interestingly, the PLDP mice showed increases in the duration of stay and number of entries into the central zone (*p* = 0.001 and 0.009, respectively) (Fig. 3E and F). These results suggested that ingestion of PLDP reduced anxiety. No significant differences were observed in grooming frequency between the two groups (*p* = 0.4173) (Fig. 3G).

**Fig. 4:**
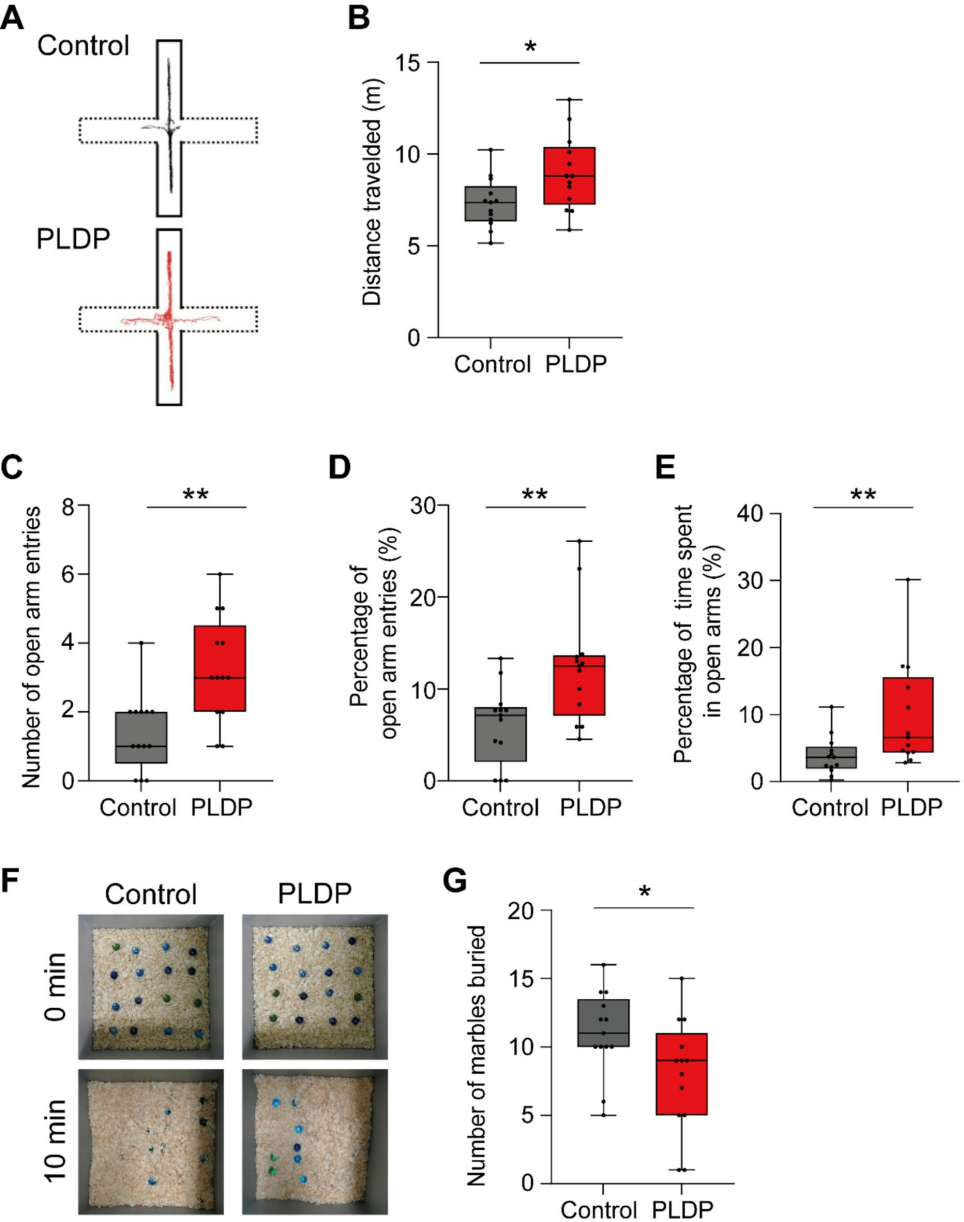
Ingestion of PLDP reduces anxiety-like behaviors. (**A–F**) Elevated plus maze test. (**A**) Representative trajectories of the PLDP and control mice. (**B**) Distance traveled, (**C**) number of entries into open arms, (**D**) percentage of open arm entries, and (**E**) percentage of time spent in open arms within 10 min. (**F** and **G**) Marble burying test. (**F**) Representative images captured before and after the test of the PLDP and control mice. (**G**) Number of marbles buried after a 10-min test. The data are represented as boxplots. The horizontal line in each box shows the median, the box shows the IQR and the whiskers are 1.5 × IQR. n = 13 in each group. **p < 0.01, *p < 0.05; Mann–Whitney’s U–test.

Next, we examined anxiety-like behaviors using the standard and simplified elevated plus maze test^18^. This test is based on the innate aversion of mice to open and elevated areas (Rodgers and Dalvi, 1997). The total travel distance of the PLDP mice was longer than that of the control mice (*p* = 0.0295) (Fig. 4A and B). The PLDP mice exhibited a significant increase in both the number and percentage of open arm entries compared to control mice (*p* = 0.0022 and 0.0059, respectively) (Fig. 4C and D). In addition, the PLDP mice exhibited a significantly higher percentage of time spent in the open arms than the control mice (*p* = 0.0072) (Fig. 4E). Subsequently, we performed a marble-burying test to evaluate anxiety-related and obsessive–compulsive-like behaviors^19,20^. The PLDP mice buried a significantly smaller number of marbles compared to the control mice (*p* = 0.0314) (Fig. 4F and G). Collectively, these results suggested that the ingestion of PLDP reduces anxiety-like behaviors.

### Ingestion of PLDP exerts antidepression effect in mice

**Fig. 5:**
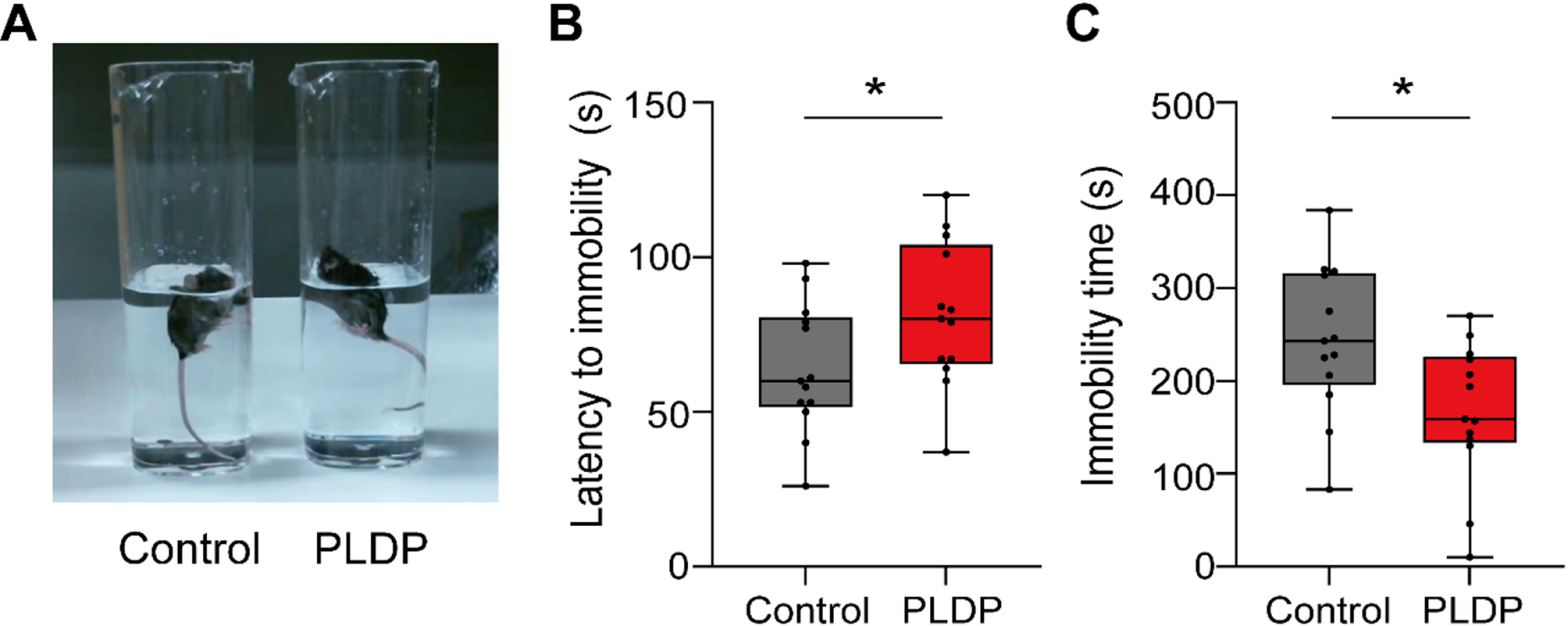
Ingestion of PLDP exerts antidepression effect. (**A**) Representative photos of the PLDP and control mice in the forced swim test. (**B**) Latency to immobility. (**C**) Immobility time during a 10-min period. The data are presented as boxplots. The horizontal line in each box shows the median; the box shows the IQR; and the whiskers are 1.5 × IQR. n = 13 in each group. *p < 0.05; Mann–Whitney’s U–test.

Next, we performed the forced swim test^21^ to evaluate the antidepressant effect of PLDP ingestion, because anxiety and depression are closely related emotion^22^. The PLDP mice exhibited a longer latency to initial immobility and reduced immobility time than the control mice (*p* = 0.0221 and 0.0373, respectively) (Fig. 5A–C). These results suggest that PLDP has an antidepressant effect.

### Ingestion of PLDP alters gene expression in the brain

**Fig. 6:**
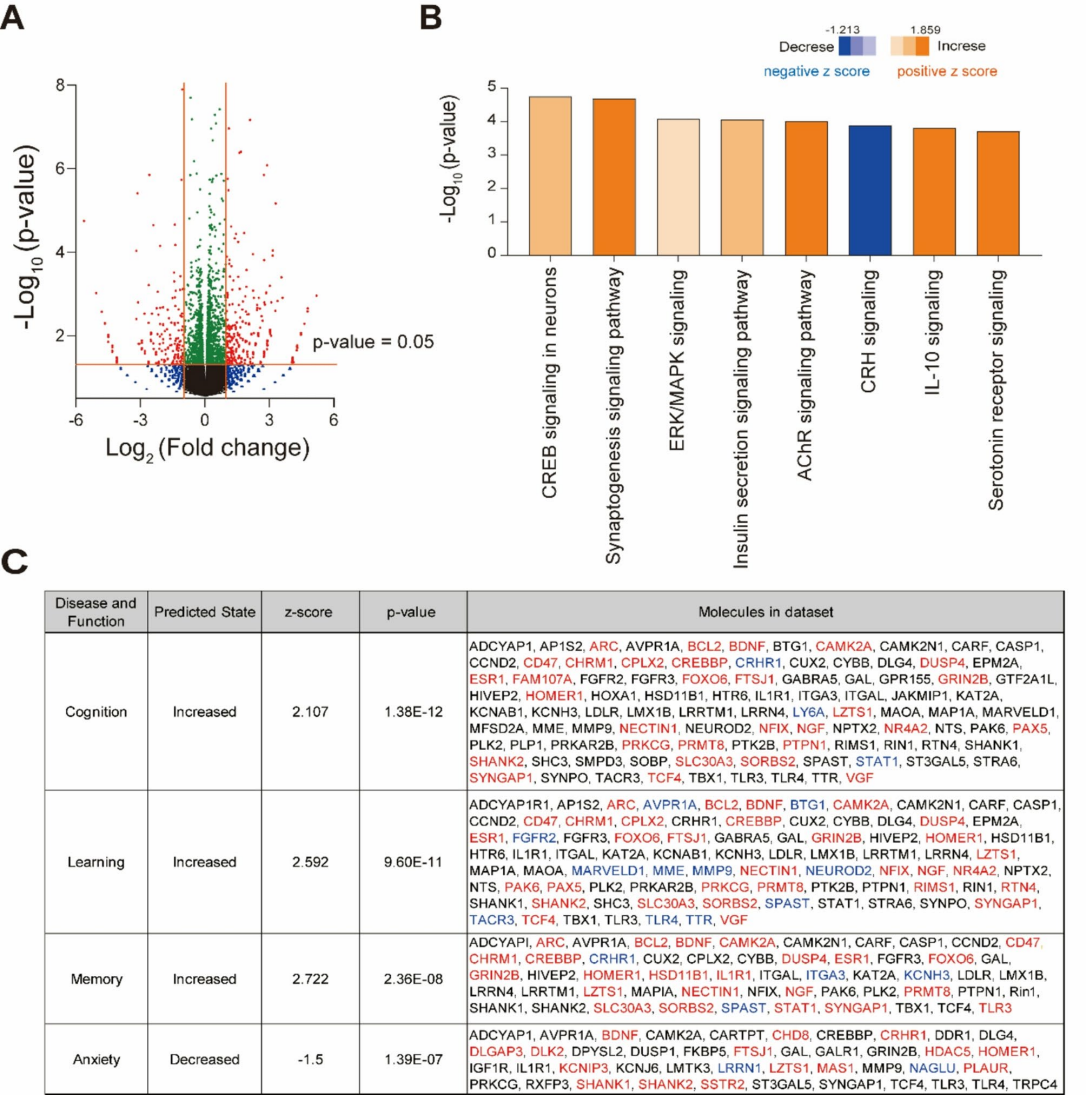
Long-term ingestion of PLDP alters gene expression in the brain. (**A**) Volcano plot showing differentially expressed genes in the brains of PLDP and control mice. Genes in PLDP mice with expression levels that significantly increased by more than twofold or decreased to less than half are shown in red. Genes in PLDP mice with expression changes within the range of 0.5-fold to 2.0-fold, showing significant decreases or increases, are shown in green. (**B**) IPA canonical pathway analysis. The canonical pathways related to cognitive and emotional functions with the lowest p-values are shown. Blue and orange bars indicate negative (inhibition) and positive (activation) z-scores, respectively. AChR, acetylcholine receptor; CREB, cyclic AMP response element-binding protein; CRH, corticotropin-releasing hormone; ERK, extracellular signal-regulated kinase; IL-10, interleukin-10; MAPK, mitogen-activated protein kinase. (**C**) IPA diseases and functional analysis. The relationship between gene expression and functional changes in the brain are shown. Molecules in dataset; A list of molecules associated with each function in IPA. Red characters represent genes significantly increased in PLDP mice, while blue characters represent genes significantly decreased.

Finally, we investigated the effects of long-term PLDP ingestion on gene expression in the brain by RNA-seq analysis. The PLDP mice exhibited significant changes in gene expression, with 342 genes showing more than two-fold increases, and 216 showing less than half of the expression compared to those in the control mice (Fig. 6A). Additionally, 929 genes showed significant increases in expression with relatively small fold changes, whereas 816 genes exhibited significantly decreased expression, but with a less pronounced reduction. Differentially expressed genes were analyzed using Ingenuity Pathway Analysis (IPA). IPA canonical pathway analysis predicted that signaling pathways with the potential to regulate memory, learning, and emotion are significantly altered in PLDP mice (Fig. 6B). Subsequent disease and function analysis predicted significant changes in functions related to cognition and emotion in the PLDP mice. Specifically, cognitive function was predicted to increase, with 33 out of 96 genes (as defined by IPA) showing expression patterns consistent with this enhancement. Similarly, learning ability was expected to increase, supported by 42 out of 84 genes aligning with this trend. Memory enhancement was also predicted, with 27 out of 55 genes demonstrating corresponding expression changes. Conversely, anxiety levels were predicted to decrease, as 17 out of 43 genes displayed expression patterns consistent with reduced anxiety (Fig. 6C).

## Discussion

Long-term ingestion of PLDP enhanced memory and locomotor activity while reducing anxiety-like and depressive behaviors in mice. Gene Ontology analysis of RNA-seq data provided additional insights into the molecular mechanisms underlying changes in memory, emotion, and locomotor activity. These results are consistent with our previous findings on the cognitive benefits of PLDP in humans^11–13^ and extend our understanding of its effects on behavior and brain functions. In addition, our results suggest that PLDP could be an effective supplement for improving cognitive health across different species.

Long-term ingestion of PLDP enhanced memory in Barnes maze test (Fig. 2). PLDP comprises protease-treated proteins, fats primarily composed of phosphatidylcholine (PC), and trace components such as vitamins (Table 1). Regarding PC, previous studies by other groups have shown its effects on cognitive functions. For example, the daily administration of 100 mg of egg-derived PC was demonstrated to improve memory in dementia model mice but not in normal mice^23^. Similarly, the intake of a 2% egg-derived PC diet (a diet containing 2% PC) improved memory acquisition and retention in memory-deficient mice but not in normal mice^24^. In contrast, a diet containing 5% egg-derived PC was reported to improve the learning ability of normal mice^25^. These previous studies by other groups suggest that the effects of PC on cognition may depend on both the baseline cognitive status and the dosage administered. The PLDP-containing diet contains approximately 0.4% liver-derived PC by weight, resulting in a daily intake of 12 mg (Tables 1 and 2, and Fig. 1). This dosage is substantially lower than the quantities demonstrated to enhance memory in previous studies conducted on mice. Consequently, it is unlikely that this amount alone would exert a memory-enhancing effect. Thus, these findings indicate that the cognitive and emotional advantages of PLDP may not be exclusively due to PC. Instead, they may arise from a synergistic interaction among various components, including peptides and other bioactive substances.

The primary components of PLDP, excluding carbohydrate, are protease-treated proteins (Table 1). Interestingly, studies by other research groups have shown that specific dipeptides and tripeptides improve cognitive performance; multiple studies have demonstrated that the oral administration of these small peptides improves cognitive performance in mouse models with cognitive impairments^26–31^. Additionally, it has been reported that some peptides exhibit anxiolytic and antidepressant effects in mice^32–35^. Therefore, the effects of the PLDP-containing diet on memory and emotional functions may be due to small peptides from protease-treated proteins. The PLDP-containing diet also enhanced locomotor activity, as observed in the open field test, Y-maze test, and elevated plus maze test (Figs. 2, 3, 4). This contrasts with previous reports from other research groups showing that small peptide ingestion alone did not affect locomotor activity^26,28,31–33,35^. Further study is needed to determine which components of PLDP contribute to the enhancement of cognitive and emotional functions, as well as the increase in locomotor activity.

The RNA-seq analysis provided insights into the molecular mechanisms underlying the observed behavioral changes. IPA canonical pathway analysis predicted that long-term PLDP ingestion positively influences cyclic AMP response element-binding protein, extracellular signal-regulated kinase/mitogen-activated protein kinase, and acetylcholine receptor signaling (Fig. 6B), which are important for memory and learning^36–39^. These predictions offer a plausible explanation for the observed enhancements in memory and learning as demonstrated in the Barnes maze test (Fig. 2). In relation to emotional regulation, IPA canonical pathway analysis predicted that PLDP negatively impacts corticotropin-releasing hormone signaling, a key regulator of the stress response implicated in mental disorders such as anxiety and depression^40,41^. Additionally, it predicted positive effects on serotonin receptor signaling, interleukin-10 (IL-10) signaling, and insulin signaling. Serotonin receptor signaling is closely associated with depression and anxiety disorders^42^. Notably, mice that overexpress IL-10 exhibit reduced depressive-like behaviors, while female IL-10 knockout mice display increased depressive-like behaviors^43^. Administration of IL-10 has also been shown to reverse depression-associated learning and memory deficits in mice^44^. Furthermore, insulin is known to enhance learning and memory and influence neurobehavioral conditions such as anxiety and depression^45^. IPA disease and function further predicted an enhancement in cognition, learning, and memory, along with a reduction in anxiety in PLDP mice (Fig. 6C). The variations in the associated genes with these predictions would further provide insights into the mechanisms underlying the changes observed in the behavioral experiments.

In this study, PLDP was administered for a duration of 16 weeks prior to the behavioral testing period, but it was not administered during the testing itself. This presents a potential limitation in the interpretation of the behavioral outcomes, as certain effects of PLDP may not have been detected due to its complete elimination from the body by the time testing commenced. Furthermore, depending on its rate of elimination, the impact of PLDP may have diminished throughout the testing period, potentially leading to variations in effect detection between the initial and final behavioral assessments. Additionally, RNA-seq analysis was conducted after the behavioral assessments, utilizing brain tissue from the same animals. At that point, PLDP administration had already been discontinued, raising the possibility that certain gene expression changes initially induced by PLDP were no longer detectable. Another limitation is that the RNA-seq analysis was performed using whole-brain samples, which may have masked region-specific molecular changes. Future studies using systematic approaches such as spatial transcriptomics or immunohistochemical analyses will be useful in clarifying these region-specific effects. Additionally, this study was conducted exclusively on male mice, and thus sex-dependent responses to PLDP were not evaluated. Future studies should address potential sex differences to provide a more comprehensive understanding of PLDP’s effects.

In conclusion, our study demonstrated that long-term ingestion of PLDP significantly enhanced memory and reduced anxiety- and depression-related behaviors in mice. These findings suggest the potential of PLDP as a natural supplement to support cognitive function, which is consistent with the results of our previous clinical trials^11–13^. Although improvements in anxiety- and depression-related behaviors were observed in mice, it remains to be determined whether these effects can be replicated in humans. Future studies should aim to confirm the reproducibility of these effects in humans and identify the specific active components of PLDP responsible for them.

## Materials and methods

### Food preparation

PLDP is a protease-treated product derived from the porcine liver^12^. The capsules containing PLDP were provided by Sugar Lady Cosmetics Co., Ltd. (Tokyo, Japan), and the contents of these capsules were used in the present study. Preparation method of PLDP is disclosed in Japan patent No. JP2016-135757A. Briefly, porcine liver was homogenized into a paste using a homogenizer or mincer. Following the addition of water, a proteolytic enzyme was applied and incubated. Subsequently, the enzyme was heat-inactivated, and the mixture was filtered to remove undigested materials. Finally, dextrin was incorporated as an excipient, and the resulting powdered mixture was encapsulated.

PLDP utilized in this study were sourced from commercially available functional food products, which are produced using by-products from animals processed for human consumption. These materials were procured and managed in accordance with ethical standards concerning the humane treatment of livestock and the efficient use of biological resources.

The components of the contents of PLDP were analyzed at Bureau Veritas F.E.A.C. Co., Ltd. (Shimane, Japan). Trace ingredients, such as minerals and vitamins, were analyzed using a different lot of PLDP from the one used in this study at the Japan Food Analysis Center (Tokyo, Japan). The PLDP-containing and control diets were prepared by Oriental Yeast Co., Ltd. (Tokyo, Japan). The PLDP-containing diet consisted of modified AIN-93M (Oriental Yeast Co., Ltd.) and 20% PLDP, while the control diet consisted of modified AIN-93M with an adjusted total caloric content (Table 2).

### Behavioral tests

All behavioral tests were performed on male C57BL/6NCrSlc mice (SLC, Tokyo, Japan). In this study, we aimed to assess the impact of PLDP ingestion on behavior. To eliminate the uncertainty surrounding the influence of PLDP on physiological variations, we used only male mice, thereby controlling for the potential confounding effects of estrous cycle variability. Sixteen-week-old mice were divided into two groups: one group was fed a PLDP-containing diet (PLDP mice: n = 13), and the other was fed a control diet (control mice: n = 13). After 16 weeks on their respective diets, the mice underwent a series of behavioral experiments. Food intake and body weight were measured every alternate week. Behavioral tests were conducted over a 17-day period following 16 weeks of dietary intervention. The schedule was as follows: open-field test (Day 1 and 2), elevated plus maze test (Day 3), forced swim test (Day 4), marble burying test (Day 5), Y-maze test (Day 6), Barnes maze test (Days 7–17: training on Days 7–11, first probe test on Day 12 [24 h after training], second probe test on Day 17 [6 days after training]). Total RNA was extracted from brains on Day 18. During the behavioral testing period, the mice did not receive the PLDP-containing diet.

### Barnes maze test

Spatial learning and memory were assessed using the Barnes maze test, essentially as previously described^46^. The apparatus consisted of a white circular platform 1.2 m in diameter with 12 holes equally spaced around its perimeter. An escape box was placed beneath the escape hole in the maze. Prior to the test, mice were habituated for three trials to familiarize themselves with the maze and escape box. During the test, two different pictures were placed in contrasting positions in a test room to provide visual assistance. The trial ended when a mouse entered the escape box or after 5 min elapsed. Training tests were conducted three times a day for five consecutive days, followed by probe tests conducted at 24 h and 6 days after the final training session to evaluate both recent and remote memory. No escape boxes were installed during the probe tests. Escape latency and time spent in the quadrant of each animal were recorded for 5 min.

### Y-maze test

Working memory was assessed using the Y-maze test, essentially as previously described^47^. The Y-maze consisted of three arms (50 × 10 × 15 cm) that diverged at an angle of 120° from the central point. Each mouse was placed in the central area. Mice were randomly placed in one of three arms and allowed to move freely into any of them. Each of the three arms was labeled with a symbol, and the number of entries into the arms and alternations were recorded. Spontaneous alternation behavior was defined as the entry into all three arms in a consecutive sequence. The percentage of alternations was calculated as the proportion of alternations to the number of alternation opportunities. The distance traveled, number of entries, and correct alternation of each animal were recorded for 8 min.

### Open field test

Locomotor activity and anxiety-like behaviors were assessed using the open field test as previously described^48^. Briefly, each mouse was placed in a corner of a square open-field apparatus (50 × 50 × 50 cm) and allowed to freely explore the arena for 30 min. Each mouse was placed in the corner of the apparatus. The center area was defined as a square 25 cm from the wall. The distance traveled, vertical activity (quantified by number of rearing), immobility time, time spent in the central zone, number of entries into the central zone, and grooming of each animal were recorded for 30 min.

### Elevated plus maze test

Anxiety-like behavior was assessed using the elevated plus maze test, essentially as described previously^46^. The test was performed using a four-arm apparatus comprising two open (30 × 5 cm) and two closed (30 × 5 × 15 cm) arms. Mice were placed in the center of maze (central platform), facing the closed arm. The distance traveled, time spent in the open arms, and number of open arms for each animal were recorded for 10 min. If a mouse fell, the test was not conducted for one trial and was repeated after the mouse had calmed down for 15 min.

### Marble burying test

Anxiety-like behavior was assessed using a marble burying test, essentially as described previously^49^. Mice were placed in a cage (25 × 25 × 25 cm) with 16 (4 × 4) evenly distributed marble balls (20 mm diameter) on top of approximately 5 cm thick sawdust bedding for 10 min. Marbles were disinfected with alcohol and dried before use. The number of marbles that were hidden and analyzed was counted. Marble balls exposed to less than or equal to 1/3 of their original area were considered to be buried, and the number of buried marble balls was blindly counted. We observed the number of marbles that a mouse hid within 10 min and analyzed the results.

### Forced swimming test

Depression-like behavior was assessed using the forced swimming test, essentially as described previously^21^. Mice were placed individually in a cylindrical glass tank (diameter × height = 16 × 30 cm) filled with water (24–26 °C) to a depth of 14 cm. Immobility time and latency to immobility time for each animal were recorded for 10 min.

### RNA sequencing analysis

RNA-seq analysis was performed using three mice from each group that were subjected to behavioral tests. After all behavioral tests had been completed, total RNA was extracted from the brains of the mice using the TRIZOL reagent (Thermo Fisher Scientific, Waltham, USA), according to the manufacturer’s instructions. Samples were subjected to TruSeq Stranded mRNA library preparation (Illumina) and sequenced on a NovaSeq 6000 (2 × 150 bp; Illumina, San Diego, CA, USA) for a 6-Gb coverage. In total, 2303 differentially expressed genes (1,271 upregulated and 1,032 downregulated genes) with *p*-values < 0.05 were further analyzed using Qiagen Ingenuity Pathway Analysis (IPA) software (QIAGEN, Valencia, CA, USA).

### Behavioral data analysis

Behavioral image data were analyzed using Prism v.9.0 software (GraphPad Software, Inc, CA, USA).

### Statistical analysis

The normality of data distribution was assessed using the Shapiro–Wilk test. Statistical significance was evaluated using two-way repeated measures ANOVA for data that met the assumption of normality. For non-normally distributed data, the non-parametric Mann–Whitney U test was used. All analyses were performed using GraphPad Prism v.9.0 (GraphPad Software, Inc.). Statistical significance was assumed when *P* < 0.05.

## Data Availability

The RNA-seq datasets generated in this study have been deposited in the NCBI Gene Expression Omnibus (GEO) repository under the accession number **GSE289386** and are accessible at [https://www.ncbi.nlm.nih.gov/geo/query/acc.cgi?acc=GSE289386] (https://www.ncbi.nlm.nih.gov/geo/query/acc.cgi?acc=GSE289386).
